# Effects of neuromuscular electrical stimulation, laser therapy and LED therapy on the masticatory system and the impact on sleep variables in cerebral palsy patients: a randomized, five arms clinical trial

**DOI:** 10.1186/1471-2474-13-71

**Published:** 2012-05-15

**Authors:** Lilian Chrystiane Giannasi, Miriam Yumi Matsui, Sandra Regina de Freitas Batista, Camila Teixeira Hardt, Carla Paes Gomes, José Benedito Oliveira Amorim, Isabella de Carvalho Aguiar, Luanda Collange, Israel dos Reis dos Santos, Ismael Souza Dias, Cláudia Santos de Oliveira, Luis Vicente Franco de Oliveira, Mônica Fernandes Gomes

**Affiliations:** 1 Department of Biosciences and Oral Diagnosis, School of Dentistry, São Paulo State University, São José dos Campos, SP, Brazil; 2 Sleep Laboratory, Nove de Julho University, Sao Paulo, SP, Brazil

## Abstract

**Background:**

Few studies demonstrate effectiveness of therapies for oral rehabilitation of patients with cerebral palsy (CP), given the difficulties in chewing, swallowing and speech, besides the intellectual, sensory and social limitations. Due to upper airway obstruction, they are also vulnerable to sleep disorders. This study aims to assess the sleep variables, through polysomnography, and masticatory dynamics, using electromiography, before and after neuromuscular electrical stimulation, associated or not with low power laser (Gallium Arsenide- Aluminun, =780 nm) and LED (= 660 nm) irradiation in CP patients.

**Methods/design:**

50 patients with CP, both gender, aged between 19 and 60 years will be enrolled in this study. The inclusion criteria are: voluntary participation, patient with hemiparesis, quadriparesis or diparetic CP, with ability to understand and respond to verbal commands. The exclusion criteria are: patients undergoing/underwent orthodontic, functional maxillary orthopedic or botulinum toxin treatment. Polysomnographic and surface electromyographic exams on masseter, temporalis and suprahyoid will be carry out in all sample. Questionnaire assessing oral characteristics will be applied. The sample will be divided into 5 treatment groups: Group 1: neuromuscular electrical stimulation; Group 2: laser therapy; Group 3: LED therapy; Group 4: neuromuscular electrical stimulation and laser therapy and Group 5: neuromuscular electrical stimulation and LED therapy. All patients will be treated during 8 consecutive weeks. After treatment, polysomnographic and electromiographic exams will be collected again.

**Discussion:**

This paper describes a five arm clinical trial assessing the examination of sleep quality and masticatory function in patients with CP under non-invasive therapies.

**Trial registration:**

The protocol for this study is registered with the Brazilian Registry of Clinical Trials - ReBEC RBR-994XFS

**Descriptors:**

Cerebral Palsy. Stomatognathic System. Electromyography. Transcutaneous Electric Nerve Stimulation. Phototherapy. Sleep Disorders. Polysomnography.

## Background

Cerebral palsy (CP) refers to disorders of motor development, rising from the primary brain injury, are permanent and changeable character, causing secondary musculoskeletal abnormalities and limitations in activities [1]. Recent studies estimate the prevalence of this condition in 2,4 per 1000 children, which means a significant number of people with this kind of disturbances [2].

Currently, the children with CP are classified according to their functional independence in gross motor function. Through Gross Motor Function Classification System (GMFCS) for CP [3], the classification is by age (0–2, 2–4, 4–6, e 6–12 years), in five functional levels. The system goal is to classify gross motor function with emphasis on the movements of "sit" and "walk" [4-6]. Children who have motor function problems similar to those classified at level 1, can generally walk without restrictions but tend to be limited in some of the more advanced motor skills. Children classified at level 5, are usually very limited in their ability to move even with the use of assistive [6].

It is known that the main alteration present in children with CP is the motor impairment, which causes several modifications rose from encephalopathy, with consequent changes in body biomechanics. In addition, the child may have intellectual disorders, sensitive, visual and hearing, which added to the motor changes, task constraints and the environment, have repercussions in different ways in their functional performance [3,7-11].

Besides the difficulties in locomotion previously described, as a result of the lack of motor coordination, orofacial alterations are also common in these individuals. These disorders are followed by pain, joint noise and irregular or deviated jaw function [12]. Individuals with spastic muscles present severely compromised function due to a diminished range of motion, diminished voluntary strength, and increased joint stiffness [13].

In general, important functions such as mastication, speech and swallowing are compromised, due to tongue thrusting, cheeks and lips incompetence, resulting in salivary incompetence, presence involuntary bite reflex, asymmetric positioning of the neck, making it difficult to maintain the posture of the head, as well as lack of dynamic balance of the masticatory muscles [14-16]. Some therapies may be suggested to treat muscular alteration in CP, such as electrical stimulation, LED therapy and laser therapy [17,18]. Neuromuscular electrical stimulation has been proposed as a potentially useful modality for muscle strengthening in children with CP. The electrical neuromuscular stimulation, it is the application of electrical stimulation of sufficient intensity to produce a visible muscle contraction which is applied to the motor point of the muscle, in order to promote muscle strengthening. However, none study that analyzed and compared their effectiveness in adult patients with CP was found so far. Considering the relation between the function of masticatory muscles and the craniofacial complex, the electromyographyc analysis (EGM) is an important tool for the undestanding of muscular pattern when developmental and functional alterations are present [19]. Evaluating and treating patients with special needs requires a multidisciplinary approach. In this context, it is important to consider that individuals with CP are also predisposed to sleep-disordered breathing, such as obstructive sleep apnea (OSA), which is one of the most common respiratory disorders. Besides, it could occur oxyhemoglobin desaturation, altered sleep-wake cycle, insomnia, disruption of sleep architecture, thus, resulting in hypoxia events during sleep. Patients with CP have a higher prevalence ranging between 50–60% of sleep-disordered breathing, when compared to individuals without CP. Health professionals should then consider the obstruction of the upper airway during wakefulness and sleep in these patients, since in most cases, OSA is not diagnosed [20-22]. In addition, sleep disorder leads to an impairment on mood, behavior, and neurocognitive function and, along with pre-existing problems in patients with PC, causes greater damage in their quality of life [20]. The measurement of sleep quality and the evaluation of sleep disorders in patients with PC are very important for the assessment of these individuals holistically, and should be added to the protocol for treating these patients. There is much to clarify about the physiology of the impact of sleep and its disorders, both in normal subjects and in patients with special needs. Forward studies are needed to search for an effective treatment protocol for improvement of quality of life of these individuals.

## Aims and hypotheses

This study aims to assess the sleep variables and masticatory dynamics by means of PSG and EMG, respectively, prior and after neuromuscular electrical stimulation, associated or not with low power laser irradiation (Gallium Arsenide- Aluminun, = 780 nm) and LED (= 660 nm) in patients with cerebral palsy. It is expected that Laser e LED biostimulation will promote the morphophysiologic recovering of muscle fibers and the decreasing of inflammatory process that will be observed through the achievement of muscular physiology within normal patterns established in this study. It is hypothesized that oxyhemoglobin desaturation, caused by pauses in breathing during sleep, can lead to harmful function in neuromuscular system in individuals with CP. We also hypothesize that electrical stimulation, led therapy and laser therapy will contribute to balance the muscular function, adjusting to physiologic patterns of muscular activity, in rest and isometric positions [17,18]. The sample will be divided according the randomization rules, in 5 groups with 10 patients (G1,G2,G3,G4,G5). In the G1 will be applied the electrical stimulation, in the G2 laser therapy, in the G3 led therapy, in the G4 the association of led therapy and electrical stimulation and in the G5 the association of laser therapy and electrical stimulation.

## Methods/design

**Figure 1:**
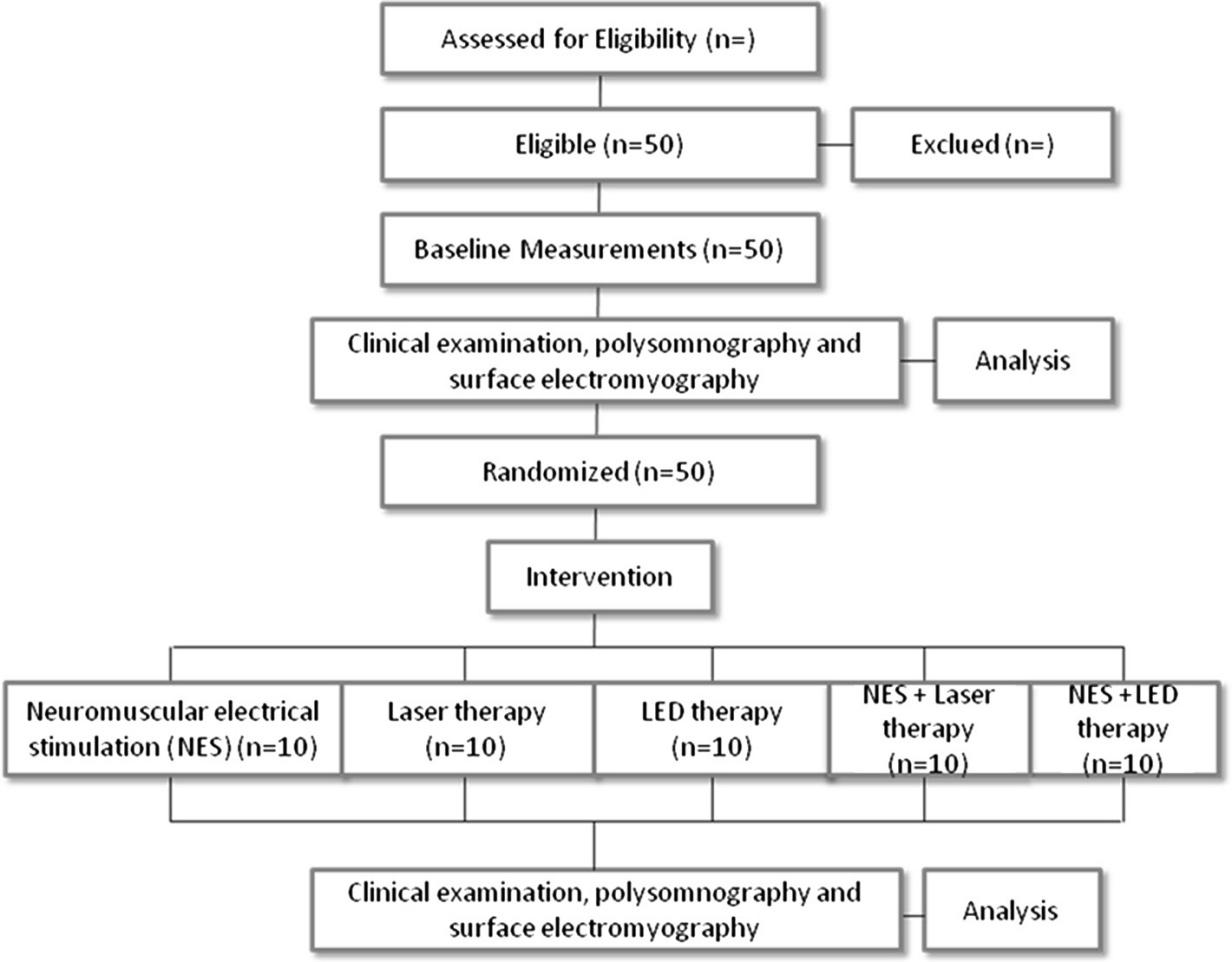
Flowchart of the study protocol.

This is a randomized, five arms clinical trial [Figure 1] conducted according to the ethical standards established in the 1961 Declaration of Helsinki (as revised in Hong Kong in 1989 and in Edimburgh, Scotland in 2000). This study is registered with the World Health Organization Universal Trial Number (UTN) U1111-1123-7969, and Registro Brasileiro de Ensaios Clínicos (RBR-994xfs), and has been approved by the Human Research Ethics Committees of the Universidade Estadual Julio de Mesquita Filho, Sao Jose dos Campos, Brazil (process number 25000.058696/2010-74). All caregivers gave written, informed consent.

## Subjects

Adult individuals between 19 and 60 years old with CP will be recruited from the Training Program in Dentistry for Persons with Disabilities, Department of Biosciences and Oral Diagnosis, School of Dentistry, São Paulo State University, Sao Jose dos Campos, SP, Brazil.

It will be included hemiparetic, quadriparetic or diparetic CP subjects, with partially preserved cognitive function, ability to respond to verbal commands, and informed consent signed by patient or patient’s responsible to voluntary participate in the study. The exclusion criteria are patients underwent to orthodontic or functional maxillary orthopedic treatment and therapies to reduce spasticity (eg. botulinum toxin) at least 6 months before the study.

## Randomization

After the evaluation of the eligibility criteria, the subjects will be randomly distributed into the five intervention groups. Randomization numbers will be generated using envelopes which will contain a card stipulating to which group the subject will be allocated. It will be used sealed and opaque envelopes to ensure confidentiality.

## Sample size

The sample size, obtained by means of statistical power analysis revealed that with 10 subjects in each group, an 90% power to detect a clinically relevant difference would be present at the alpha level of 0.05.

## Study interventions

### Clinical evaluation of oromotor functions

Anamnesis will be obtained in order to assess chief complaint, onset, frequency, evolution of the problem, consulted professionals, treatments, results and prescription of drugs, medical and family history, parafunctional habits and psychogenic aspects.

A specific part of the questionnaire will approach sleep breathing disorders, including snoring, choking during sleep, drooling, nightmare experience, movement during sleep and mood after waking. For the clinical examination, dental occlusion, tooth wear, tooth loss, Mallampati evaluation [23] and tonsils classification [24] will be evaluated. Subjects will also be classified according to the five levels of Gross Motor Function Classification System [3]. It is emphasized that oropharyngeal alterations presented in patients with CP will be reviewed by a speech therapist. A modified scale of orofacial motor function assessment for adults with CP, based on Santos [25], will be used, in order to evaluate oral motor function, by performing simple movements such as coordination and performance of voluntary facial muscles, jaw protrusion and lateral movement, tongue movements, such as elevation and laterality, lip muscle strength (puff-out cheeks/maintain pressure), glossopharyngeal/hypoglossal motor activity and rapid coordinated jaw, lip, tongue and palatal movement. According to the ability of the patient to perform properly or not each movement, it will be applied the score 0 (inability to perform the movement) to 2 (ability to complete the movement). The sum of scores for each item will result in the total value of the patients oromotor function.

### Surface electromyography

**Figure 2:**
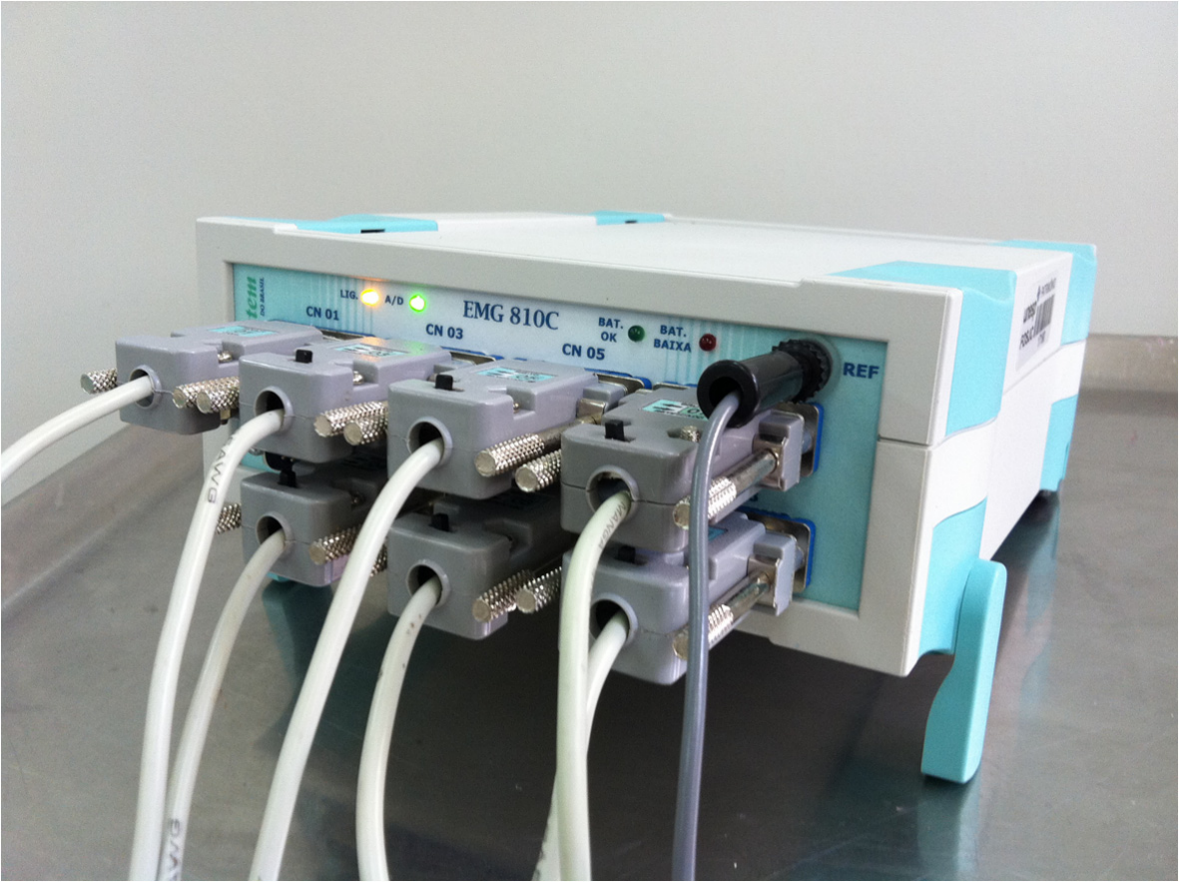
Electromyography equipment used in this study.

**Figure 3:**
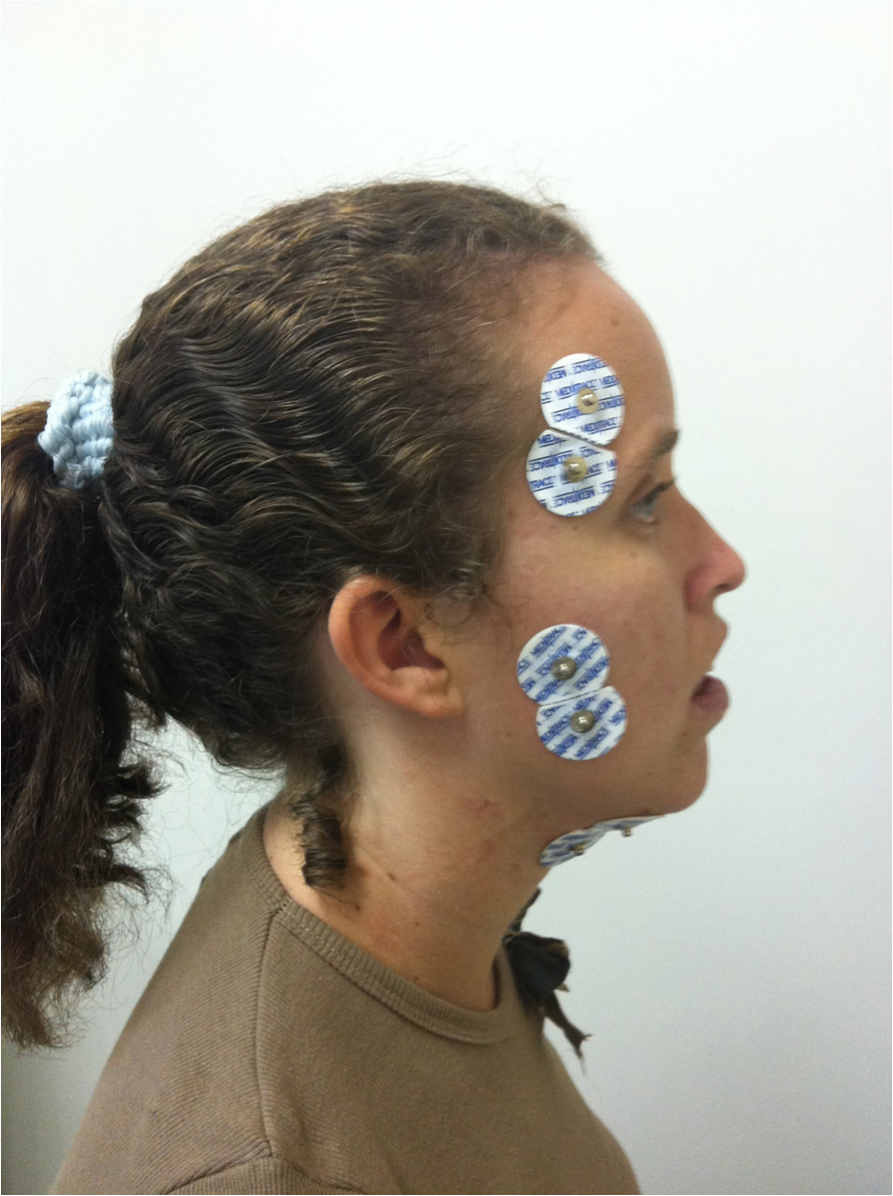
Electrodes placement.

For the EMG record, it will be used an eight-channel electromyography equipment (EMG-800 C, EMG System of Brazil Ltda, Sao Jose dos Campos, SP) [Figure 2, previously calibrated with amplification of 2000 times and 16-bit resolution. Six input channels will be used to assess the following muscles: channel 1 - anterior portion of right temporalis muscle, channel 2 - superficial portion of right masseter; channel 3 - anterior portion of left temporalis muscle; channel 4 - superficial portion of the left masseter, channel 5 - right suprahyoid muscles; and channel 6 - left suprahyoid muscles. The other two channels will be used for the force transducer and mandibular goniometer. Bipolar, small, passive, circular and disposable Ag/AgC surface electrodes (Meditrace® Kendall-LTP, Chicopee, MA) will be used for evaluation of masticatory system activity. A reference electrode will be positioned in the patients right wrist to reduce undesirable interferences of the electromyographic signal. Volunteers will remain seated, with natural head position during sEMG exam. Electromyographic signals will be recorded after cleaning the skin with 70% alcohol to reduce skin impedance and to allow proper placement of surface electrodes. Surface electrodes will be bilaterally placed according to anatomical references and procedures guided by the direction of muscle fibers in three points, the anterior temporal muscle – 2 to 3 cm superior-posterior distant to the lateral corner of the eyes in the region of greatest evidence of muscle mass, no hair, parallel to the muscle fibers, but with its sensing surface perpendicularly oriented; the superficial portion of masseter – 1 to 2 cm above the gonial angle of the mandible, in the region of greatest evidence of muscle mass, with muscle fibers parallel to the surface, and supra-hyoid muscles – in the region of greatest evidence of muscle mass, parallel to the muscle fibers [26] [Figure 3.

### Analysis of sEMG data

The average data will be expressed in RMS (Root Mean Square) which qualitatively expresses the record of electrical activity of muscles under study [27].

### Protocol for electromyographic examination

**Figure 4:**
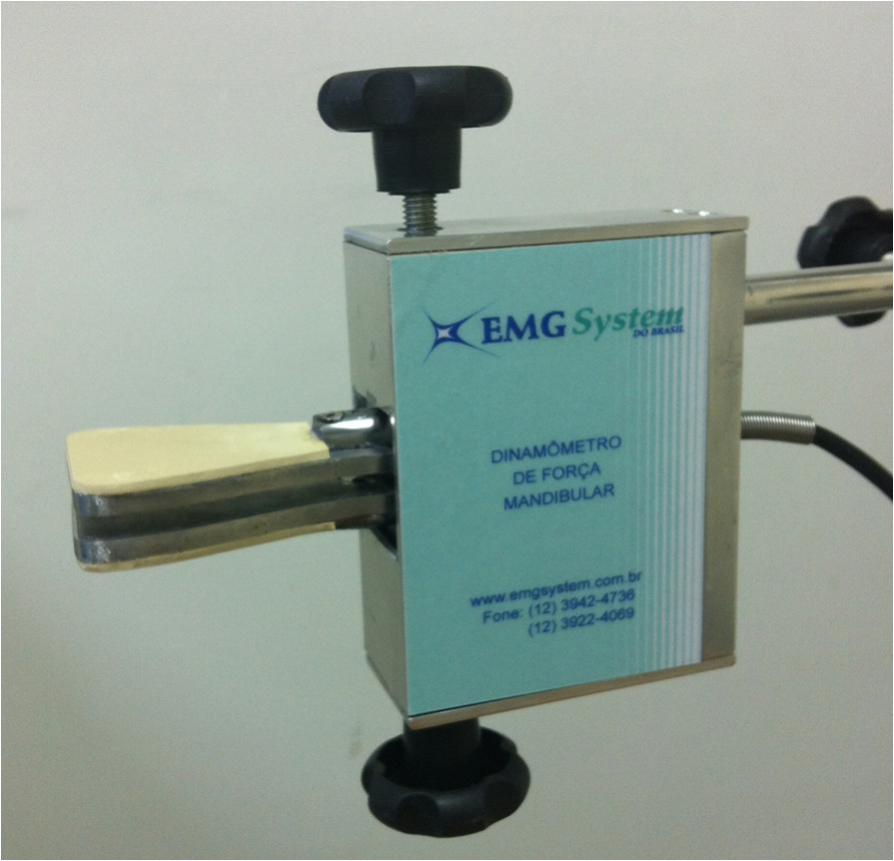
Mandibular force transducer equipment used in this study.

**Figure 5:**
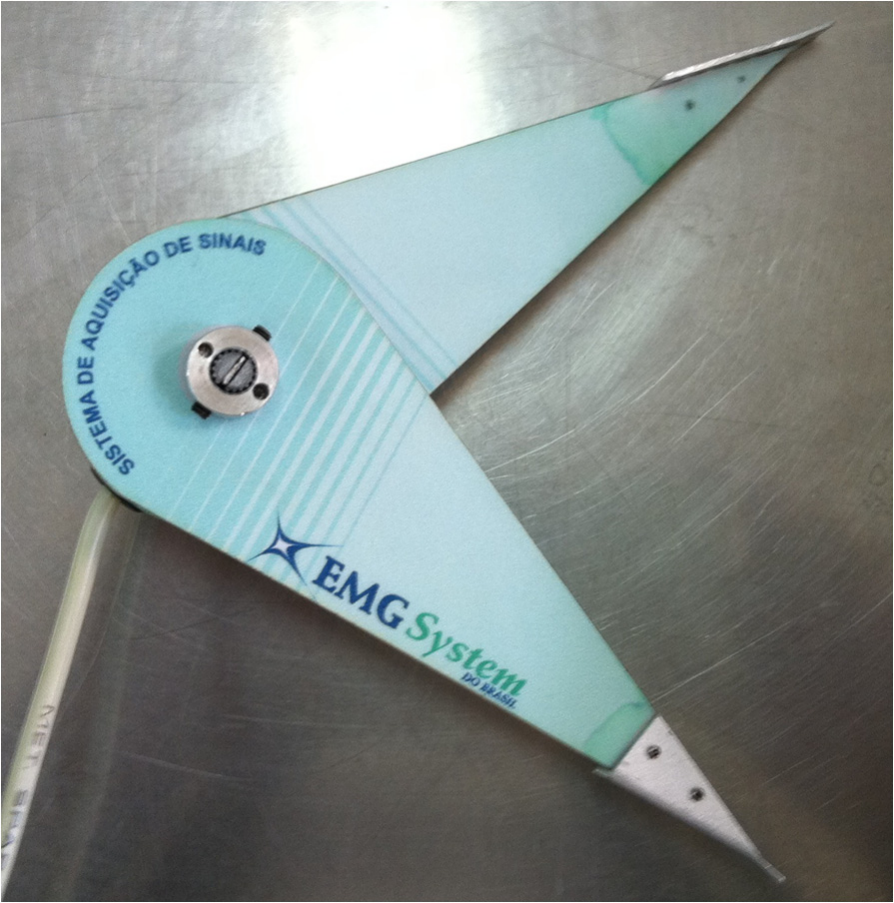
Mandibular goniomenter equipment used in this study.

It will be used a mandibular force transducer (EMG System of Brazil Ltda, Sao Jose dos Campos, SP) [Figure 4 to record the maximum bite force, which consists of a mechanical device with sensors that record material deformations during the bite. This deformation is converted into kgf or Newton by means of EMGLab V1.1 software (EMG System of Brazil Ltda). In order to measure the mouth opening amplitude, it will be adopted a mandibular goniometer (EMG System do Brazil Ltda, Sao Jose dos Campos, SP) [Figure 5. The electromyographic recordings will be performed in all phases of the study, described below, in the rest position, isometric position, bite force, using a transducer, and opening/closing with the aid of the mandibular goniometer. Each EMG recording will last ten seconds with an interval of one minute and will be repeated three times at the same appointment [28].

### Phases of electromyographic exam

The sEMG, referring to the masseter, temporallis, supra-hyoid, bite force and range of mandibular opening shall be provided in four distinct phases. Phase 1- initial data collection (Groups 1 to 5); Phase 2- treated groups (Groups 1 to 5) after 1 week of electrical stimulation with or without laser and LED therapy; Phase 3-treated groups (Groups 1 to 5) after 4 week of electrical stimulation with or without laser and LED therapy and Phase 4-treated groups (Groups 1 to 5) after 8 weeks after the last electrical stimulation with or without laser and LED therapy.

Data obtained will be compared among each group to verify the effectiveness of the proposed therapies to improve the masticatory muscle activity in patients with CP.

### Protocol for laser and LED therapy

After the evaluation and diagnosis, patients will be randomly divided into 5 groups (n = 10). Groups 2 to 5 will be treated with lasertherapy or LED theraphy, combined or not with electricalstimulation twice a week for eight consecutive weeks, following the protocol of [29].

The craniofacial complex will be irradiated in 12 areas, being 1. posterior region of the temporomandibular joint (TMJ) with open mouth, reaching the auriculo temporal nerve; 2. area prior to the sigmoid notch, insertion area of the lateral pterygoid muscle (upper beam) at the neck of the condyle and disk; 3. articular interface between condyle and fossa with open mouth; 4. angle of the jaw; 5. anterior temporal muscle; 6. middle portion of the temporal muscle; 7. posterior portion of the temporal muscle; 8. upper, middle and bottom of the sternocleidomastoid muscle; 9. anterior portion of occipitofrontal muscle; 10. posterior portion of the occipitofrontal muscle; 11. superficial portion of the masseter; and 12. supra-hyoid muscles. In groups 2 and 4, these anatomical structures will be irradiated with a laser diode of gallium arsenide and aluminum-GaAlAs (TWIN Laser, Optics brand MM), emitting at a wavelength of 660 nm, with a constant power 40 mW, and a maximum beam diameter of 0.38 cm^2^. Groups 3 and 5 will be irradiated with a light emitting diode (LED), emitting a wavelength band of 630 ± 5 nm, with a constant power 40 mW, and the maximum laser beam diameter of 0.38 cm^2^. Both will be operated in continuous mode and should be used in contact with the target tissue, providing an irradiance or intensity of 0.40 mW/cm^2^. The incidence of fluency range for each point of application will be of 12.0 J/cm^2^, and the irradiation time of 30 seconds for each predetermined point.

### Protocol for neuromuscular electrical stimulation (NMES)

NMES is a noninvasive technique, without systemic effects, is not addictive and has no undesirable side effects. This technique consists on the application of mild electrical stimulation through electrodes placed on the surface of muscles. It induces action potentials in motor nerve, causing activation of motor units [30]. Effects such as strengthening the stimulated muscles, facilitation of voluntary motor control [31] and decreased spasticity have been reported.

Neuromuscular electrical stimulation (Neurodyn III) equipment will be used. In this study, a protocol will be applied based on Nunes [32] recommendations, which are sessions of 30 minutes (divided between the superficial portion of masseter, the anterior portion of temporalis muscle and supra-hyoid, according to the electromyogram diagnosis), 2 times per week for 8 weeks compatible with a total of NMES 16 sessions in patients of Groups 1, 4 and 5. After 8 weeks of NMES training, both neural and muscular adaptations mediate the strength improvement .

### Protocol for polysomnography

A full-night PSG [33] will be performed prior and after all therapies, using a digital system (Embla, A10 version 3.1.2 Flaga, Hs. Medical Devices, Iceland) at the Sleep Laboratory of University of Nove de Julho. All recording sensors will be attached to the patient in a non-invasive manner using tape or elastic bands. The following physiological variables will be monitored simultaneously and continuously: four channels for the electroencephalogram (EEG) (C3-A2, C4-A1, O1-A2, O2-A1), two channels for the electrooculogram (EOG) (EOG-Left-A2, EOG-Right-A1), four channels for the surface electromyogram (muscles of the submentonian region, anterior tibialis muscle, masseter region and seventh intercostal space), one channel for an electrocardiogram (derivation V1 modified), airflow detection via two channels through a thermocouple (one channel) and nasal pressure (one channel), respiratory effort of the thorax (one channel) and the abdomen (one channel) via x-trace belts, snoring (one channel) and body position (one channel) via EMBLA sensors, and arterial oxygen saturation (SaO2) and pulse rate via an EMBLA oximeter. All PSGs will be performed and sleep stages visually scored according to standardized criteria for investigating sleep. EEG arousals, sleep-related respiratory events and leg movements will be scored in accordance with the criteria established by the American Academy of Sleep Medicine Manual for Scoring Sleep and Associated Events [34]. The patients will be instructed to remain as relaxed as possible and sleep naturally, as if at home. All signals will be recorded continuously. Throughout the night, all the subjects will be monitored by a technician experienced in polysomnography [33].

### Quality control

In order to ensure data quality, dentists in charge of EMG exam, as well as the speech-therapist in charge of oral movements and sleep technician in charge of the data acquisition of polysomnography will receive specific training. Periodic external monitoring will be performed to verify the adequate polysomnnographic examination. The results of the preoperative and postoperative exams will be analysed by blinded evaluators.

### Statistical analysis

Data will be presented as means ± standard deviation, when applicable. For comparison of continuous variables prior and after polysomnography and specifics therapies, it will be used the paired Student *t*-test or Wilcoxon tests as appropriate. Comparisons between groups will be performed using Student *t* test or Mann–Whitney U according to the distribution. All tests will be 2 tailed, and p values of less than 0.05 will be assumed to represent statistical significance. All analyses will be performed using SPSS ver. 16.0.

## Discussion

This study will evaluate the effects of lower power laser, neuromuscular electrical stimulation, LED therapy, as well as neuromuscular electrical stimulation plus LED therapy and neuromuscular electrical stimulation plus laser therapy on the masticatory muscles activity in adults with CP, by means of surface electromyography. We believe that electrical stimulation may act in the modulation of muscle hyperactivity/hypoactivity, adjusting them to a level close to normality. Also, we expect the LED and laser morphophysiological favor recovery, which will be observed clinically by the absence or reduction of pain. In addition, polysomnography will be used to evaluate the sleep variables and to detect sleep-breathing disorders, and we believe that the sleep quality will be improved after therapies application.

## Competing interests

The authors declare that they have no competing interests.

## Authors’ contributions

All the authors contributed to the conception and design the study. LCG and MFG provided the idea for the study, established the hypothesis and wrote the original proposal. SRFB is a speech therapist of the patients and made a contribution to evaluate and collect the data of the oral motor function. LCG, MYM, CTH and CPG made a contribution to acquisition and interpretation of EMG data. IRS, EFO, and ISD made a contribution to acquisition and interpretation of PSG data. LCG and LC significantly contributed to statistical analizys. SRN is a medical doctor, sleep specialist, made a contribution to literature research and shall make polysomnographics reports. ICA and JBOA were involved in critically revising the manuscript. CSO, LVFO and MFG supervised this study, participated in its design and coordination and, revised the manuscript that led to the final approval of the current submission. All authors read and approved the final manuscript.

## Pre-publication history

The pre-publication history for this paper can be accessed here:

http://www.biomedcentral.com/1471-2474/13/71/prepub
